# Recruiting participants for interventions to prevent the onset of depressive disorders: Possibile ways to increase participation rates

**DOI:** 10.1186/1472-6963-10-181

**Published:** 2010-06-25

**Authors:** Pim Cuijpers, Annemieke van Straten, Lisanne Warmerdam, Marie José van Rooy

**Affiliations:** 1 Department of Clinical Psychology, VU University Amsterdam, The Netherlands; 2 EMGO Institute for Health and Care Research, VU University Amsterdam and VU University Medical Center, The Netherlands; 3 ZonMw, Netherlands Organisation for Health Research and Development, The Hague, The Netherland

## Abstract

**Background:**

Although indicated prevention of depression is available for about 80% of the Dutch population at little or no cost, only a small proportion of those with subthreshold depression make use of these services.

**Methods:**

A narrative review is conducted of the Dutch preventive services in mental health care, also addressing the problem of low participation rates. We describe possible causes of these low participation rates, which may be related to the participants themselves, the service system, and the communication to the public, and we put forward possible solutions to this problem.

**Results:**

There are three main groups of reasons why the participation rates are low: reasons within the participants (e.g., not considering themselves as being at risk; thinking the interventions are not effective; or being unwilling to participate because of the stigma associated with depression); reasons within the health care system; and reasons associated with the communication about the preventive services. Possible solutions to increasing the participation rate include organizing mass media campaigns, developing internet-based preventive interventions, adapting preventive interventions to the needs of specific subpopulations, positioning the services in primary care, integrating the interventions in community-wide interventions, and systematically screening high-risk groups for potential participants.

**Discussion:**

Prevention could play an important role in public mental health in reducing the enormous burden of depression. However, before this can be realized more research is needed to explore why participation rates are low and how these rates can be improved.

## Introduction

It has long been thought that it is not feasible to prevent the onset of depressive disorders, because the processes involved in the etiology are too complex and not yet sufficiently understood. A growing number of randomized controlled trials have shown, however, that it is possible in some cases to prevent or at least delay the onset of depressive disorders. A recent meta-analysis of these studies showed that the risk of developing a depressive disorder in people who received a preventive intervention was 22% lower than those who did not receive such an intervention [1]. In the past 30 years a considerable infrastructure for preventive services has been developed in the Netherlands. Most ambulatory mental health services have a prevention department serving the local or regional community. One important service aimed at the prevention of depressive disorders consists of psychoeducational group interventions. These interventions are based on the psychoeducational "Coping with Depression" course which has been proven to be effective in the prevention of depressive disorders [2]. The courses are aimed at people with subthreshold depression, and participants are recruited directly from the general population. It is estimated that about 80% of the Dutch population has direct access to these preventive services at little or no cost.

Only a fraction of the people with a subthreshold depression participates in these preventive courses, despite their wide availability. Subthreshold depression can be defined as clinically relevant depressive symptoms that do not meet the full criteria for a depressive disorder. In this paper, we will explore the problem of low participation rates, analyzing the causes and examining possible solutions. We show how research can be of help in establishing which solutions are most effective. Although this is a national issue, we think it is also important from an international perspective, because it is a problem which may occur in other countries as well when preventive services aimed at depression are disseminated. As far as we know, the Netherlands is the only country to have made preventive services of this nature available to the general population.

### Recruiting participants for preventive services in mental health

The "Coping with Depression" preventive courses (CWD) are organized at the local level in the Netherlands by the prevention departments of the regional ambulatory mental health services. Participants are recruited through media announcements and via referrals from health professionals. In the media announcements, the intervention is presented as a way to learn mood management skills in a psychoeducational setting with 'teachers' and 'students'. It is not registered at a national level how many of these preventive courses are organized each year and how many people participate. However, a national registry of participants in preventive services for depression showed that in 2007 a total of 8,273 people participated in a preventive intervention for depression (of whom 3,263 participated in an online preventive intervention) [3].

It can be assumed that this is an important benefit from the viewpoint of the participants, because these courses result in significant reductions in depressive symptomatology and a considerable reduction in the risk of developing a depressive disorder (the chance of developing a depressive disorder is 38% lower than in care-as-usual control groups) [2].

However, from a public health perspective, the impact of these courses is small. It is estimated that about 7.5% of the Dutch adult population suffered from subthreshold depressive disorder in the past year [4]. With a population of 10 million people aged between 18 and 65, this suggests that about 750,000 people in this age group suffer from subthreshold depression each year. The total number of participants in the CWD courses is about 1 percent of this group.

Importantly, many participants in the CWD courses in routine practice suffer from conditions other than subthreshold depression and therefore do not belong to the target group. The CWD courses are intended as indicated prevention, aimed at people with subthreshold depression, to prevent the onset of major depression according to diagnostic criteria [5]. A recent study among older participants of the CWD in the Netherlands, showed that according to a diagnostic interview based on the DSM-IV, 39% had a major depressive disorder (MDD), and 45% had an anxiety disorder [6]. This implies that a considerable number of participants have a major depression and that these participants consider the courses to be a treatment of their disorder. Accordingly, the number of people with subthreshold depression reached by the current CWD courses is probably even lower than the 1 percent cited above.

### Possible causes

Unfortunately, there is very little research on the causes of these low participation rates, and the possibilities we put forward in this paragraph are largely speculative. The low level of participation in preventive services for mental health may be related to: (1) unwillingness in people with subthreshold depression to participate, (2) the organizational structure in which the courses are embedded, and (3) the recruitment methods of the interventions. In this paragraph we will examine these three possible reasons.

#### 1. Unwillingness to participate

This may be due to the following factors:

- the stigma associated with mental disorders;

- the belief that such services are not effective;

- they do not see themselves as having subthreshold depression and in need of a preventive intervention (this could be the case in people who for example are recently divorced, who have lost their partner through death, or who are caring for a sick relative; they know they have problems, but would not consider this to be depression, and may not see a need for a mood management intervention aimed at the prevention of depression);

- other reasons (such as the group format of these interventions which assumes that participants are willing to share their problems with others; or coinciding commitments at the time of the sessions).

Many people with a major depressive disorder do not seek treatment (about 40% of sufferers in the Netherlands) [7], and help-seeking is associated with more severe depression and the presence of comorbidity. Depressed people have several reasons for not seeking treatment. Many indicate that they would rather solve the problems themselves; they think treatment does not work; they do not know where to get help; are afraid to ask for help; or do not have the money to pay for treatment [8]. It would seem logical therefore that people with subthreshold depression are even less inclined to seek help than those with major depression, and even less so than those with more severe depressive disorders.

There is one study on public attitudes towards prevention of depression [9]. This study showed that the majority of a representative population sample in Germany (75.4%) was positively disposed toward the possibility of preventing depression, and that half of them expressed willingness to take part in prevention programs. This suggests that the attitude of the public towards prevention of depression is positive, and that the main reasons for not participating in preventive services are not related to this attitude.

#### 2. Causes associated with the organizational structure of the courses

- Currently, preventive services are organized within mental health care. It is entirely possible that this set-up has a limiting effect on the familiarity with these services among potential participants, or among professionals who can refer people to these services.

- Most prevention departments only have limited capacity to organize prevention interventions, and it is entirely possible that if more capacity and resources were available, the total number of participants would also increase.

- There may be obstacles that hinder professionals in referring patients to these courses

▪ they may not be aware of the existence of these interventions,

▪ they may not recognize the subthreshold depression in their patient,

▪ they may doubt the evidence base of the interventions,

▪ or they may be insufficiently aware of the inclusion criteria and the possible benefits of the courses.

#### 3. Causes associated with the recruitment methods

There could well be many people with subthreshold depression who might be willing to participate in these services, but are unaware of the possibility of doing so. As indicated above, this may be related to insufficient knowledge among professionals, such as general practitioners, about these services.

Participants in CWD courses are usually recruited through announcements in local media and through referrals by health professionals. If this communication is not adequate, the target population may not be aware of the existence of these services.

### Possible ways to increase the participation rates in preventive services

There are several possibilities to increase the participation rates in preventive services.

#### 1. Solutions aimed at potential participants

- Mass media campaigns to reduce the stigma associated with mental disorders could significantly boost participation rates, as well as media campaigns stressing the feasibility of preventing the onset of depressive disorders. Several mass media campaigns aimed at depression and mental disorders have been conducted, including the "Defeat Depression" and "Changing Minds" campaigns in the United Kingdom [10-12], the "Depression, Awareness, Recognition and Treatment" and "National Depression Screening Day" campaigns in the United States [13-15], "Beyondblue" in Australia [16,17], and the "Nuremberg Alliance Against Depression" and "European Alliance Against Depression" in Europe [18,19]. Whether these campaigns have actually resulted in increased participation rates has, however, not yet been well established.

- Offering the CWD courses through the Internet may also help to reduce stigma and encourage people to make use of these services. Internet-based interventions not only have been shown to be as effective as face-to-face interventions [20,21], but they save therapists' time, eliminate traveling time, reduce waiting-lists, cut costs, allow people to work at their own pace, abolish the need to schedule appointments, avoid the stigma of going to a therapist, and they may reach people who are not willing to participate in face-to-face interventions [22].

- Tailoring the CWD to specific subgroups of participants could also improve participation. Interventions aimed at specific subgroups may encourage potential participants to actually participate. In the past years, several versions of the CWD have been developed for specific target groups, for example older adults, adolescents, and people with a chronic general medical disorder. However, it is also possible to develop versions for people with specific medical disorders, for people from ethnic minority groups, or people from lower socioeconomic groups. These more specific interventions can be applied through the Internet, individually or in groups.

- Because comorbidity between depressive and anxiety disorders is very high, it may also be worthwhile to focus preventive interventions on depression as well as anxiety. It is entirely possible that interventions aimed at depression will also prevent the onset of anxiety disorders, and vice versa [23]. Combining both approaches may also result in larger groups for preventive services.

#### 2. Organizational solutions

- If these services were located in primary care, it is likely that general practitioners would refer many more patients than they currently do. Most depressed patients are treated in primary care [7], and it can be assumed that many patients with subthreshold depression who could benefit from preventive services are also known to the general practitioner. The results of studies examining the effects of preventive interventions in primary care are quite promising [24-26].

- We have already referred to the use of the Internet in the delivery of prevention programs, the possibility of using the courses in an individual format for people who are not willing to participate in group interventions, and the possibility of increasing the resources available for these preventive services.

- Currently, preventive courses with a fixed schedule are offered at fixed dates. It may also be possible to organize them more flexibly for example, in modular format.

- Community interventions have been successful in several areas of preventive healthcare, including alcohol prevention [27,28], smoking cessation [29], and general mental health problems [30], although few such initiatives aimed at preventing depression are available. Community interventions are organized within a specific community, and both professionals and community members decide which interventions will be offered.. Preventive courses could well be integrated into such interventions, and this may lead to an increased participation rate.

#### 3. Improving recruitment methods

- We have already mentioned the possibility of mass media campaigns aimed at reducing the stigma associated with mental disorders, and campaigns designed to increase awareness of the evidence supporting the possibility of preventing depression. Mass media campaigns could also be used to direct attention to the interventions themselves, and to increase awareness in the community of the existence of these services. Because most mass media campaigns are very costly, more resources are needed to utilize this possibility.

- Another way of recruiting participants is through systematic screening of populations. Several studies have shown that systematic screening of adolescents at school and offering a preventive intervention to those with subthreshold depression is effective in reducing the incidence of major depression [31,32]. The same is true for screening and intervening in pregnant women at risk for developing postpartum depression [33-35].

Summarizing, the most important possibilities to increase participation rates in preventive mental health services are:

#### Aimed at potential participants

- Media campaigns to reduce the stigma associated with mental disorders

- Media campaigns stressing that it is possible to prevent the onset of depressive disorders, and indicating who are at risk

- Adapt the CWD courses (further) to specific subgroups of potential participants

#### Organizational solutions

- Offer the CWD through the Internet

- Offer the CWD as an individual or modular intervention?

- Increase resources for organizing CWD courses

- Position the preventive services in primary care instead of in mental health care

- Embed CWD courses in broader community interventions

#### Communication

- Increase awareness in health professionals about preventive services

- Increase resources for recruitment

- Conduct systematic screening of potential participants

### How can research help improve participation rates in preventive interventions

There are several important research questions that should be addressed in order to improve participation rates in preventive interventions. First, there is very little knowledge about why participation rates are so low. Most possibilities to improve participation rates described earlier are not based on empirical evidence. More research among people with subthreshold depression is needed to examine the exact reasons for not participating. It would also be very useful to ask participants who do participate what their reasons are for participating. This could be used to improve communication about the services. Research comparing the socio-demographic characteristics of participants to the characteristics of the population with subthreshold depression could show where there are gaps in program delivery, and where efforts to increase participation could be focused. Furthermore, studies are needed that examine the knowledge and attitude of professionals who may refer potential participants to these services.

Research on mental health prevention lacks connection with the theoretical developments in physical health promotion. Theories of behavior change developed in this field, such as the "Health Belief Model" [36], or the "Theory of Planned Behavior" [37,38] could provide guidance in understanding why indicated prevention programs for depression are not being taken up.

Another worthwhile area of research concerns the adaptation of the interventions to specific target groups. It is well established that the CWD is effective, but not whether it remains effective when it is adapted to other types of interventions or to other target groups. For example, although the CWD has been adapted for Internet application [20,21], no trial has examined whether this has resulted in a significant reduction of new incidences of depressive disorders. Related to this issue is the question whether further adaptation of preventive interventions will indeed lead to improved access to these services.

A third area of research should focus on implementation studies. We have seen that there are several organizational models for providing preventive services. Also, there are several methods to improve communication about the interventions to the target groups. It might well be possible to conduct randomized community trials in which communities are randomly assigned to different models to provide these services. The most important outcome of interest would be the number of participants in the preventive interventions in the different models.

A fourth area of research should focus on preventing depression in combination with other mental disorders. For example, the comorbidity of depression and anxiety disorders is very high, and it may be possible to develop preventive interventions for depression, which are also effective as prevention of anxiety disorders, and vice versa. If we could combine different preventive interventions with each other, it may well be possible to increase the impact of these, and to recruit more people with different risk profiles into the same interventions. However, to date there is no research supporting or even examining this hypothesis.

## Conclusions

Although indicated preventive services aimed at the prevention of depressive disorders are available for the majority of the Dutch population at little or no cost to participants, only a small proportion of people with subthreshold depression participate. In the current article we described this problem, presented an overview of possible causes, and we proposed solutions to increasing access to these services.

As far as we know, the Netherlands is the only country in the world where preventive services aimed at the prevention of depression are available at low cost to the general population. It may be possible that the low participation rates are related to the culture in the Netherlands, and if these services were offered in other countries, the participation rates would be much higher. However, this does not seem very likely.

Before widespread dissemination of indicated prevention of depression is possible, we have to answer the question why so few people participate. As we have seen in this paper, the answer to that question is not yet clear. It is encouraging, however, that prevention of new cases of depressive disorders seems to be feasible and effective. As an adjunct to treatment, prevention may play an important role in public mental health to reduce the enormous burden of depression. The development of evidence-based prevention of depression and other mental disorders ought to be an important scientific and public health objective for the 21st Century.

## Competing interests

The authors declare that they have no competing interests.

## Authors' contributions

All authors were involved in the design and content of the paper and have given final approval of the version to be published. PC wrote the drafts of the texts and the revisions.

## Pre-publication history

The pre-publication history for this paper can be accessed here:

http://www.biomedcentral.com/1472-6963/10/181/prepub
